# Scrotal Abscess Mimicking Fixed Drug Eruption Under Ibuprofen Therapy: A Diagnostic Pitfall

**DOI:** 10.1002/ccr3.72549

**Published:** 2026-04-24

**Authors:** Youssef Maachi, Amine ELBoustani, Amine Salim Lalaoui, Amine Slaoui, Tariq Karmouni, Abdelatif Koutani, Khalid Elkhader

**Affiliations:** ^1^ Department of Urology B, CHU Ibn Sina, Faculty of Medicine and Pharmacy Mohamed V University Rabat Morocco

**Keywords:** anchoring bias, diagnostic error, fixed drug eruption, ibuprofen, NSAIDs, scrotal abscess, scrotal ultrasound

## Abstract

Concurrent NSAID therapy can pharmacologically mask early scrotal abscess, generating a presentation that fulfills clinical and historical criteria for fixed drug eruption. Ibuprofen simultaneously acts as a recognized causative agent and suppresses infectious inflammatory signs. Normal inflammatory markers and absent fluctuation do not exclude an abscess. Early scrotal ultrasound is mandatory.

**Key Message**

Concurrent NSAID therapy can pharmacologically mimic a presentation of early scrotal abscess that fulfills clinical and historical criteria for fixed drug eruption. Normal inflammatory markers and absent fluctuation do not exclude an abscess under NSAIDs. A scrotal ultrasound must be performed early in any scrotal erythema unresponsive to initial treatment.

## 1 Introduction

Scrotal erythema is a deceptively polymorphic presentation whose differential diagnosis spans from benign and self‐limiting conditions—contact dermatitis, tinea cruris, fixed drug eruption—to surgical emergencies such as Fournier's gangrene. The clinical challenge lies not only in distinguishing these entities but in recognizing how concurrent medications may alter the presentation of infectious processes, creating diagnostic mimicry that delays appropriate treatment.

Fixed drug eruption (FDE) is a type IV delayed hypersensitivity reaction mediated by resident memory CD8+ T lymphocytes that accumulate at specific cutaneous sites upon initial sensitization [1]. Its defining characteristic is the recurrence of erythematous or violaceous plaques at identical anatomical locations upon re‐exposure to the causative agent [2]. Genital involvement occurs in approximately 30% to 50% of cases [3], and non‐steroidal anti‐inflammatory drugs (NSAIDs)—particularly ibuprofen—are among the most frequently implicated causative agents [4].

However, NSAIDs also exert systemic anti‐inflammatory effects through cyclooxygenase inhibition, reducing prostaglandin synthesis and attenuating the cardinal signs of infection: Fever, pain, erythema, and swelling [5]. This pharmacological action may render early scrotal abscess clinically indistinguishable from a drug reaction, particularly when the patient is receiving a drug known to cause FDE at the very site of infection.

We report a case in which this convergence—a recognized FDE‐causative drug, a prior similar episode, and NSAID‐mediated masking of infectious signs—led to a seven‐day delay in the diagnosis of early scrotal abscess, ultimately confirmed by microbiological culture. To our knowledge, this specific pharmacological mechanism of FDE mimicry by scrotal abscess has not been previously described.

## 2 Case Presentation/Examination

A 65‐year‐old man with no significant past medical history presented to the urology outpatient clinic with a five‐day history of left scrotal erythema and mild discomfort. He reported having experienced an identical erythematous plaque at the same scrotal site approximately three months earlier, which had resolved spontaneously without medical consultation or treatment.

The patient had been self‐medicating with ibuprofen 400 mg three times daily for lumbar pain for several days prior to the onset of the scrotal lesion and continued this treatment at the time of presentation.

**FIGURE 1:**
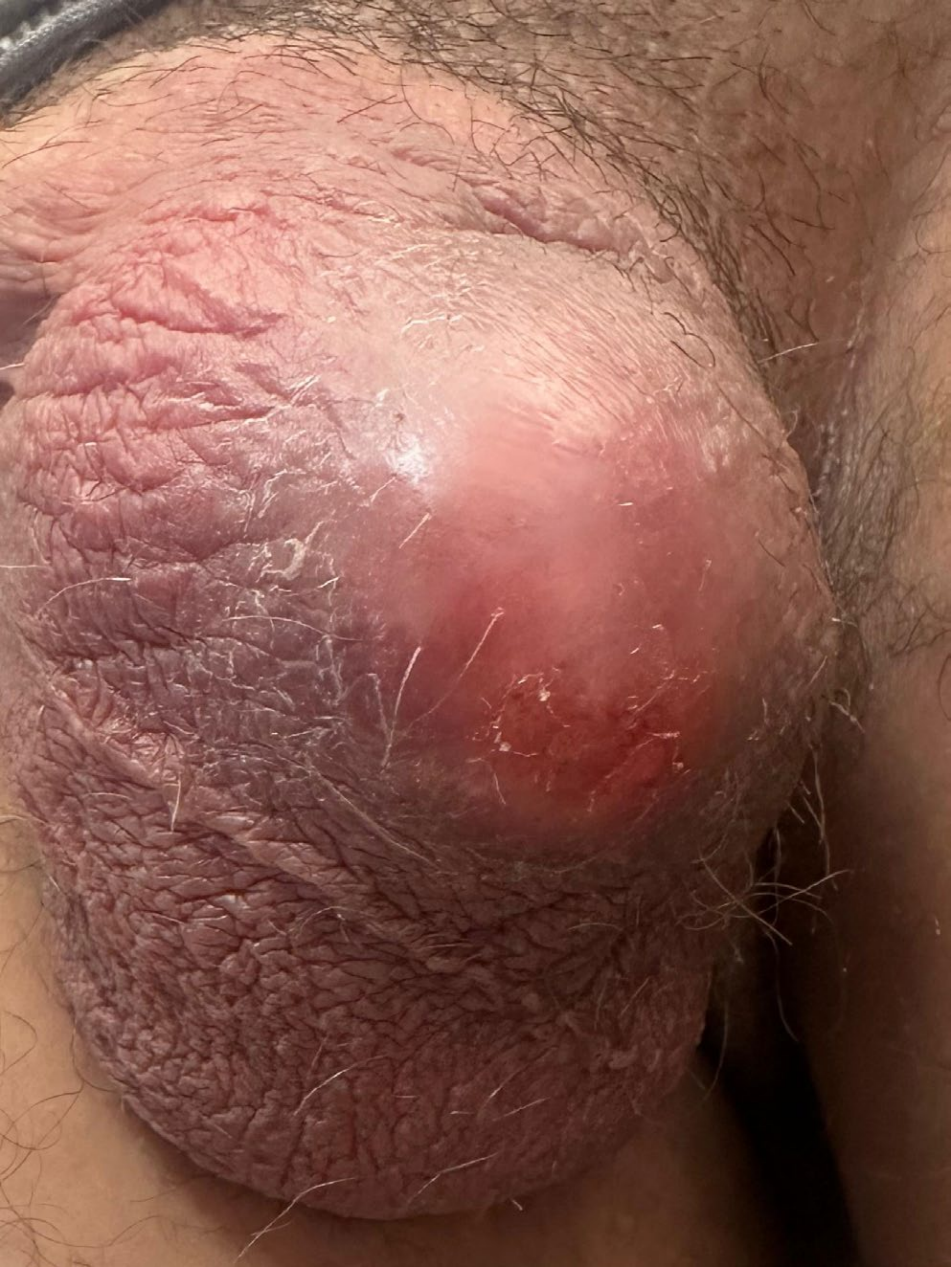
(provided as separate file): Left hemiscrotal erythematous plaque at initial presentation (Day 0). A well‐demarcated, oval, erythematous, and indurated plaque measuring approximately 4 cm is visible on the left hemiscrotum. No vesiculation, fluctuation, or satellite lesions were clinically detectable. The tense, slightly shiny central surface reflects subcutaneous inflammatory tension attenuated by concurrent ibuprofen therapy. This presentation fulfilled the clinical criteria for fixed drug eruption and was managed as such for seven days prior to diagnostic revision.

Physical examination revealed a well‐demarcated, oval, erythematous plaque measuring 4 cm on the left hemiscrotum (Figure 1). No fluctuation was clinically detectable. No vesiculation, satellite lesions, or regional lymphadenopathy were noted. Body temperature was 37.1°C. Discomfort was mild and partially controlled by ibuprofen.

Initial laboratory investigations revealed a white blood cell count of 7,200/mm³ with a normal differential, a negative C‐reactive protein, and renal and hepatic function within normal limits at the time of presentation.

## 3 Differential Diagnosis, Investigations, and Treatment

### 3.1 Initial Differential Diagnosis and Working Diagnosis

The clinical picture, a well‐demarcated erythematous scrotal plaque without systemic inflammatory signs, a prior identical episode three months earlier at the same anatomical site, and concurrent ibuprofen therapy strongly oriented the diagnosis toward fixed drug eruption. Topical betamethasone 0.05% twice daily was initiated. Ibuprofen was administered in accordance with the FDE diagnostic methodology to evaluate lesion behavior under sustained drug exposure.

A variety of illnesses were evaluated in the differential diagnosis of a solitary scrotal erythematous plaque prior to ascribing the lesion to fixed drug eruption or abscess. Folliculitis presents as superficial papulopustules centered on hair follicles, typically smaller than 1 cm and multiple; the size and depth of this lesion excluded this diagnosis. Infected epidermoid cyst characteristically presents as a pre‐existing firm subcutaneous nodule with progressive enlargement; the patient reported no prior palpable mass at the site. Hidradenitis suppurativa produces recurrent, often bilateral inflammatory nodules at apocrine‐bearing skin, typically with axillary or inguinal involvement and a chronic relapsing course; neither the clinical presentation nor the history was consistent with this diagnosis.

### 3.2 Clinical Evolution and Diagnostic Revision

During the course of the next four to six days, the expected improvement in the lesion with topical corticosteroid therapy was not observed. Instead, progressive induration was noted on successive examinations, while fluctuation remained clinically absent. The patient experienced a gradual increase in discomfort despite continued ibuprofen use. On the seventh day, the patient spontaneously presented with worsening pain, marked local warmth, and increased swelling. Re‐examination revealed marked induration and suspicion of a deep collection. Repeat laboratory tests revealed a white blood cell count of 12,000 cells per cubic millimeter and a C‐reactive protein level of 80 mg per liter.

### 3.3 Investigations

Scrotal ultrasound identified a well‐defined hypoechoic subcutaneous collection measuring 2 cm within the left scrotal wall. No involvement of the testicular parenchyma or epididymis was detected. A urological workup was performed: PSA level 3 ng/mL (within normal limits), supple prostate on digital rectal examination without nodularity or tenderness, normal urinary flow rate on uroflowmetry, and prostate volume of 42 g on ultrasound consistent with mild benign prostatic hyperplasia without significant infravesical obstruction.

### 3.4 Treatment

The diagnosis was revised to scrotal abscess. Under sterile conditions, aspiration of yellowish purulent material was obtained. Pus culture revealed *Escherichia coli* , concordant with a positive urine culture (ECBU), indicating a common urogenital infectious source. Antibiotic sensitivity confirmed susceptibility to ceftriaxone. Ibuprofen was discontinued. Intravenous ceftriaxone 1 g daily was initiated as empiric treatment and continued as monotherapy for a total of seven days following antibiogram confirmation.

The complete clinical timeline, from initial presentation to final follow‐up, is summarized in Table 1.

**TABLE 1:** Clinical Timeline.

Timepoint	Clinical Events
Day 0 (Presentation)	65‐year‐old man, no significant past medical history, presents with 5‐day history of left scrotal erythema and mild discomfort. Self‐medicating with ibuprofen 400 mg TID for lumbar pain (ongoing). History of identical spontaneously‐resolved scrotal plaque 3 months prior. Examination: 4 cm well‐demarcated oval erythematous plaque, left hemiscrotum; no fluctuation, no fever (T° 37.1°C), no vesiculation, no lymphadenopathy. CBC: WBC 7,200/mm^3^. CRP: Negative. Working diagnosis: Fixed Drug Eruption (ibuprofen as suspected causative agent).
Days 1–3	FDE diagnosis maintained. Topical betamethasone 0.05% twice daily initiated. Ibuprofen continued. No systemic symptoms.
Days 4–6	Lesion fails to improve despite topical corticosteroid therapy. Progressive induration noted on palpation. Fluctuation remains clinically absent. Gradual increase in local discomfort despite ibuprofen.
Day 7 (Return visit)	Patient returns with worsening pain, marked local warmth, and increased swelling. Repeat examination: Significant induration, deep collection suspected. Repeat labs: WBC 12,000/mm^3^, CRP 80 mg/L. Scrotal ultrasound: Well‐defined hypoechoic subcutaneous collection, 2 cm, left scrotal wall—no testicular or epididymal involvement.
Day 7 (Treatment)	Diagnosis revised: SCROTAL ABSCESS. Aspiration under sterile conditions: Yellowish purulent fluid obtained. Pus culture: *Escherichia coli* . ECBU: Positive ( *Escherichia coli* —concordant). Empirical ceftriaxone 1 g IV once daily initiated. Ibuprofen discontinued. Antibiogram subsequently confirms *E. coli* sensitivity to ceftriaxone → monotherapy continued for 7 days total.
Days 8–10	Rapid clinical improvement within 48 h: Erythema, induration, and pain progressively resolved. Wound care continued.
Week 3 (Follow‐up)	Complete resolution of scrotal lesion confirmed. No recurrence. Antibiotic course completed. Mild residual skin discoloration at drainage site.

## 4 Conclusion and Results (Outcome and Follow‐Up)

### 4.1 Outcome

Clinical response was rapid: Erythema, induration, and pain resolved within three days of drainage and targeted antibiotic therapy. Complete healing was confirmed at the three‐week follow‐up with no recurrence and mild residual skin discoloration at the drainage site.

### 4.2 Conclusion

This case demonstrates that concurrent NSAID therapy can generate a pharmacologically masked presentation of early scrotal abscess that fulfills the clinical and historical criteria for fixed drug eruption. The dual role of ibuprofen as both a recognized FDE‐causative agent and a suppressor of infectious inflammatory signs creates a specific and underrecognized diagnostic trap. A prior similar episode at the same anatomical site, rather than confirming FDE, may reflect an earlier undiagnosed infectious event and should not substitute for imaging evaluation. Three practical lessons emerge from this case. First, the presence of a recognized FDE‐causative drug does not establish the diagnosis of drug eruption and does not justify deferring infectious workup. Second, normal inflammatory markers and absent fluctuation do not exclude an abscess when NSAIDs are being administered. Third, scrotal ultrasound must be performed early—not after clinical deterioration—in any scrotal erythema that fails to respond as expected to initial treatment. Microbiological confirmation through pus culture remains essential to establish the definitive diagnosis and guide antibiotic therapy.

## 5 Discussion

### 5.1 A Pharmacologically Constructed Diagnostic Trap

This case illustrates a mechanism of diagnostic error that differs from classical anchoring bias: The diagnostic hypothesis was not simply premature—it was pharmacologically manufactured. Ibuprofen simultaneously generated the conditions for FDE suspicion (recognized causative agent, prior similar episode at the identical anatomical site) and suppressed the inflammatory signs that would have otherwise indicated infection. The result was a self‐reinforcing diagnostic loop in which the drug suspected of causing the eruption was also actively concealing the actual diagnosis.

This dual pharmacological role of NSAIDs in scrotal pathology has not, to our knowledge, been previously described as a distinct mechanism of diagnostic confusion. Its recognition is clinically important because it applies to any patient receiving NSAID therapy who presents with scrotal erythema, regardless of drug history.

### 5.2 The Prior Episode: A Diagnostic Trap, Not a Diagnostic Criterion

The history of an identical scrotal plaque three months earlier, spontaneously resolved, was the element that most powerfully anchored the initial diagnosis of FDE. Anatomical recurrence at the identical site is considered a pathognomonic criterion of FDE, reflecting the persistence of sensitized resident memory CD8+ T cells at specific cutaneous locations [1, 2].

However, this case demonstrates that a history of prior scrotal erythema must be interpreted with caution. Coincidental prior episodes of scrotal inflammation—infectious, inflammatory, or undiagnosed—may create a false appearance of FDE recurrence when no microbiological or histological confirmation was obtained at the time. In this patient, the nature of the prior episode remains undetermined; it may represent an earlier self‐limited infectious episode rather than a drug reaction. The absence of hyperpigmentation at the prior site—a finding considered pathognomonic for FDE resolution—was not documented, which itself represents a missed diagnostic opportunity.

### 5.3 Histopathological Confirmation of FDE using Skin Biopsy in Ambiguous Clinical Presentation

In non‐bullous FDE, biopsy characteristically demonstrates interface dermatitis with vacuolar change, necrotic keratinocytes at all levels of the epidermis, and dermal melanophage accumulation—the latter being present in 66% of non‐bullous cases and constituting a histological hallmark of prior interface dermatitis [6]. Persistent post‐inflammatory hyperpigmentation at the resolution site reflects this melanophage accumulation and is considered a defining clinical criterion for FDE; its absence at the prior site in this patient should have prompted reconsideration of the diagnosis [7]. In the present case, neither histological confirmation nor documented residual hyperpigmentation was available for the prior episode, leaving its nature undetermined.

### 5.4 Differential Diagnosis: Excluding Other Scrotal Lesions

Several conditions must be considered in the differential diagnosis of a solitary scrotal erythematous plaque before attributing the lesion to FDE or abscess. Folliculitis presents as superficial papulopustules centered on hair follicles, typically smaller than 1 cm and multiple; the size and depth of this lesion excluded this diagnosis. Infected epidermoid cyst characteristically presents as a pre‐existing firm subcutaneous nodule with progressive enlargement; the patient reported no prior palpable mass at the site. Hidradenitis suppurativa produces recurrent, often bilateral inflammatory nodules at apocrine‐bearing skin, typically with axillary or inguinal involvement and a chronic relapsing course; neither the clinical presentation nor the history was consistent with this diagnosis [8].

In retrospect, the 4 cm size, the depth of the collection on ultrasound, and the positive microbiological cultures unambiguously established the diagnosis of scrotal abscess. None of the alternative diagnoses above would produce a 2 cm deep subcutaneous fluid collection.

### 5.5 NSAIDs As Masking Agents of Scrotal Infection

The anti‐inflammatory properties of NSAIDs—inhibition of cyclooxygenase‐1 and cyclooxygenase‐2, reduction of prostaglandin E2 synthesis, attenuation of the febrile response—are well established [5]. Their capacity to delay the diagnosis of scrotal infections has been documented in the context of Fournier's gangrene, where early stages may present with erythema and swelling indistinguishable from benign conditions, and where diagnostic delay is consistently associated with more advanced disease at presentation [9].

The scrotal region presents specific anatomical vulnerability to this masking effect. The loose areolar connective tissue of the scrotal wall allows fluid accumulation without generating the tension that would normally produce clinically detectable fluctuation. Combined with NSAID‐mediated reduction of pain and attenuation of the systemic febrile response, this anatomical feature explains why a 2 cm subcutaneous collection with a rising inflammatory response remained clinically occult for seven days in this patient.

### 5.6 Microbiological Findings and Antibiotic Selection

The isolation of *Escherichia coli* in both pus culture and urine culture established a common urogenital infectious source and confirmed the microbiological diagnosis independently of clinical findings alone. *E. coli* is the most frequently isolated organism in scrotal and perineal infections, including in the Fournier's gangrene spectrum [8]. This concordance is clinically significant: It excludes skin flora contamination as the infectious origin and confirms urogenital seeding as the pathogenic mechanism, consistent with the patient's age and the epidemiology of gram‐negative scrotal infections in older men.

The 2024 EAU Guidelines on Urological Infections identify Enterobacterales as the predominant pathogens in male accessory gland infections, and specify that in up to 90% of cases, acute scrotal infection results from pathogen migration from the urethra or bladder [10]. Accordingly, a structured urological workup is indicated in any male patient with a gram‐negative scrotal infection, including digital rectal examination to evaluate for prostatic involvement, renal and bladder ultrasound to exclude obstructive uropathy, and post‐void residual measurement to detect infravesical obstruction, a particularly relevant consideration in men over 60 years of age [10].

In this patient, urological evaluation was performed and revealed a PSA level within normal limits at 3 ng/mL, a supple prostate on digital rectal examination without nodularity or tenderness, a normal urinary flow rate on uroflowmetry, and a prostate volume of 42 g on ultrasound consistent with mild benign prostatic hyperplasia without significant infravesical obstruction. This structured workup confirms urogenital seeding as the infectious pathway in the absence of an obstructive or malignant predisposing factor and represents the recommended evaluation for gram‐negative scrotal infection in men over 60 years of age as outlined in the 2024 EAU Guidelines [10].

Furthermore, the EAU diagnostic algorithm for acute epididymitis explicitly recommends scrotal ultrasound when there is failure to respond to initial treatment or when an abscess is suspected [10], a recommendation that, in retrospect, supports earlier imaging in this case rather than awaiting clinical deterioration on day seven.

Antibiotic therapy was initiated empirically with ceftriaxone 1 g intravenously once daily, based on clinical presentation and local epidemiological patterns favoring gram‐negative coverage. Upon antibiogram confirmation of *E. coli* susceptibility to ceftriaxone, monotherapy was continued for a total of seven days. The rapid clinical response—complete resolution of pain, erythema, and swelling within three days of drainage—validated this targeted approach retrospectively. While broader empirical coverage, including anaerobic organisms (such as amoxicillin‐clavulanate or ceftriaxone combined with metronidazole), is appropriate when culture results are unavailable [11], antibiogram‐directed monotherapy with a third‐generation cephalosporin is justified when microbiological confirmation identifies a susceptible gram‐negative organism as the sole pathogen.

### 5.7 Scrotal Ultrasound: The Diagnostic Resolution

Scrotal ultrasound is the reference imaging modality for acute scrotal pathology, providing real‐time visualization of subcutaneous collections, testicular parenchyma, and epididymis with high spatial resolution and the ability to differentiate infectious from non‐infectious processes [3, 12]. In this case, ultrasound identified a 2 cm hypoechoic collection entirely undetectable on clinical examination—a direct consequence of NSAID‐mediated attenuation of inflammatory tension within the scrotal wall.

We propose that scrotal ultrasound should be performed without delay when: The lesion fails to improve after 48 to 72 h of appropriate treatment for the presumed diagnosis; NSAIDs or corticosteroids are being administered concurrently; induration progresses despite anti‐inflammatory therapy; or any clinical feature is discordant with the expected natural history of the working diagnosis. In this case, the seven‐day delay resulted from awaiting clinical deterioration rather than proactively imaging a lesion that failed to behave as expected.

Table 2 summarizes the key differentiating features between FDE and early scrotal abscess under NSAID therapy.

**TABLE 2:** Differential Diagnosis: Fixed Drug Eruption vs. Early Scrotal Abscess Under NSAID Therapy.

Feature	Fixed Drug Eruption	Early Scrotal Abscess Under NSAIDs
Morphology	Flat/slightly raised plaque	Raised, tense; fluctuation may be absent early
Borders	Well‐demarcated, sharp	May appear well‐demarcated early
Pain	Mild pruritus	Masked by NSAIDs — may be mild
Fever	Absent	May be absent under NSAIDs
WBC/CRP	Normal	May be normal early; rises over days
Prior episode	Same site — pathognomonic	Coincidental — does not confirm FDE
Response to TCS	Progressive resolution	No improvement or worsening
Ultrasound	No collection	Hypoechoic fluid collection
Pus culture	Not applicable	Positive — confirms infectious etiology
Resolution	Spontaneous + hyperpigmentation	Requires drainage + antibiotics

## 6 Conclusion

This case demonstrates that concurrent NSAID therapy can generate a pharmacologically masked presentation of early scrotal abscess that fulfills the clinical and historical criteria for fixed drug eruption. The dual role of ibuprofen—as both a recognized FDE‐causative agent and a suppressor of infectious inflammatory signs—creates a specific and underrecognized diagnostic trap. A prior similar episode at the same anatomical site, rather than confirming FDE, may reflect an earlier undiagnosed infectious event and should not substitute for imaging evaluation.

Three practical lessons emerge from this case. First, the presence of a recognized FDE‐causative drug does not establish the diagnosis of drug eruption and does not justify deferring infectious workup. Second, normal inflammatory markers and absent fluctuation do not exclude an abscess when NSAIDs are being administered. Third, scrotal ultrasound must be performed early—not after clinical deterioration—in any scrotal erythema that fails to respond as expected to initial treatment. Microbiological confirmation through pus culture remains essential to establish the infectious diagnosis and guide antibiotic therapy.

## Author Contributions

**Youssef Maachi:** conceptualization, writing – review and editing, writing – original draft, formal analysis, project administration. **Amine ELBoustani:** investigation, software. **Amine Salim Lalaoui:** data curation, methodology. **Amine Slaoui:** validation, visualization. **Tariq Karmouni:** validation, visualization. **Abdelatif Koutani:** validation, visualization. **Khalid Elkhader:** visualization, validation, supervision.

## Funding

This research received no specific grant from any funding agency in the public, commercial, or not‐for‐profit sectors.

## Ethics Statement

This case report did not require formal ethics committee approval in accordance with local and national guidelines, as it involves a single retrospective case report with no experimental intervention. All procedures performed were in accordance with the ethical standards of CHU Ibn Sina, Rabat, Morocco, and with the 1964 Helsinki Declaration and its later amendments.

## Consent

Written informed consent was obtained from the patient for publication of this case report and any accompanying images. A copy of the written consent is available for review by the Editor‐in‐Chief of this journal upon request.

## Conflicts of Interest

The authors declare no conflicts of interest.

## Data Availability

The data that support the findings of this study are available from the corresponding author upon reasonable request.
